# Establishing a social behavior paradigm for female mice

**DOI:** 10.3389/fnins.2025.1630491

**Published:** 2025-07-21

**Authors:** Hanyang Xiao, Changgang Huang, Yue Wu, Jacob Junlin Wang, Hao Wang

**Affiliations:** ^1^Department of Neurosurgery of Second Affiliated Hospital and School of Brain Science and Brain Medicine, Key Laboratory for Biomedical Engineering of Education Ministry, Zhejiang University School of Medicine, Hangzhou, China; ^2^Nanhu Brain-Computer Interface Institute, Hangzhou, China; ^3^NHC and CAMS Key Laboratory of Medical Neurobiology, MOE Frontier Science Center for Brain Research and Brain Machine Integration, Key Laboratory of Precise Treatment and Clinical Translational Research of Neurological Diseases, School of Brain Science and Brain Medicine, Zhejiang University, Hangzhou, China; ^4^Basis International School, Hangzhou, China; ^5^Lingang Laboratory, Shanghai, China

**Keywords:** social behavior, female mice, cagemate, novelty preference, three-chamber test

## Abstract

**Introduction:**

Social behavior assessment in female mice has been historically challenged by inconsistent results from the classic three-chamber test, which reliably detects social preferences in males but fails to capture female specific social dynamics.

**Methods:**

We developed a modified three-chamber paradigm by replacing standard social stimuli with familiar cagemates (co-housed for 2 weeks, 1 week or 24 hours) to better assess sociability and novelty preference in female mice.

**Results:**

In the sociability phase, female mice showed a significant preference for interacting with cagemates compared to empty chambers. Crucially, during the social preference phase, test females demonstrated robust novelty seeking behavior, spending significantly more time exploring novel conspecifics compared to 2-week cagemates or 1-week cagemates. This preference trended similarly, though non significantly, with 24-hour cagemates. Notably, our paradigm enhanced social preference indices without altering total interaction time, confirming its specificity for detecting novelty driven exploration.

**Discussion:**

These findings overcome the limitations of traditional paradigms and establish a validated framework for studying female social behavior, with critical implications for modeling neurodevelopmental disorders like autism spectrum disorder (ASD) in female preclinical research.

## 1 Introduction

Social behaviors are essential for the survival and wellbeing of many species including rodents and humans, as they facilitate cooperative and competitive interactions among conspecifics (Okada and Bingham, 2008; Hamlin, 2018; Lee and Beery, 2019). Social recognition quantification evaluates an organism’s ability to discriminate conspecifics through integrated processing of learned social signatures and innate identification mechanisms (Choleris et al., 2012). The assessment of social recognition abilities gauges the capacity of an individual to differentiate conspecifics based on information acquired from past interactive experiences or innate knowledge.

Mice are extensively utilized as model organisms for studying social functions due to their capacity to discriminate between social and nonsocial stimuli while exhibiting an innate preference for conspecific interaction (Moy et al., 2004). They can also discern familiar from unfamiliar conspecific, relying mainly on olfactory cues for social recognition (Ryan et al., 2008). Mice tend to spend more time investigating unfamiliar conspecifics compared to familiar ones, which is a basic prerequisite for making appropriate responses (e.g., approach, exploration, fighting and avoidance) in social interactions (Beery and Shambaugh, 2021; Keppler and Molas, 2024).

One of the most commonly used experimental paradigms for assessing social function in mice is the three-chamber social test. This method can reflect social willingness and social recognition abilities based on the animal’s preference for social versus nonsocial stimuli (sociability) or familiar versus unfamiliar stimuli (social preference) (Rein et al., 2020). While these tests reliably reflect social function in male mice (Nadler et al., 2004; Shoji et al., 2016), studies using this three-chamber paradigm with the female mice social task are controversial. Some studies have indicated that pubertal (5–6 weeks old) show social interest toward unfamiliar female mice (Moy et al., 2004), while other studies have found that female mice exhibit less social engagement with unfamiliar female conspecifics and display different patterns of social recognition (Karlsson et al., 2016; Bertoni et al., 2021). Bertoni et al. (2021) demonstrated that while adult female mice exhibited intact sociability, their inconsistent social recognition and short-term social memory results question the validity of the classic three-chamber test for assessing female-to-female social function.

Social dysfunction constitutes a hallmark of autism spectrum disorder (ASD) and related neurodevelopmental conditions (Young et al., 2002; Bal et al., 2019; Maisterrena et al., 2024). However, preclinical research using murine models has historically focused on male subjects (Pan et al., 2022; Yan et al., 2022; Hooshmandi et al., 2023), with female cohorts systematically excluded due to absence of validated behavioral paradigms sensitive to female specific social phenotypes. This gender gap persists despite well documented ASD prevalence in females, as demonstrated by Autism and Developmental Disabilities Monitoring Network’s data showing a 4:1 male-to-female diagnostic ratio in human populations (Maenner et al., 2023; Maisterrena et al., 2024). Thus, developing a refined behavioral paradigm to evaluate social function in female animal models is imperative for further investigating the underlying mechanisms.

## 2 Materials and methods

### 2.1 Animals

All experimental procedures were conducted according to the guidelines of Zhejiang University Animal Experimentation Committee. Adult C57BL/6 J mice (8 weeks old, RRID: IMSR_JAX:000664) were purchased from Shanghai SLAC Laboratory Animal Co., Ltd. Before the experiments, the mice were housed in groups of 4–6 per cage at the Zhejiang University Laboratory Animal Center (SPF grade) 2 weeks to acclimated our experimental environment. For the modified three-chamber test, in the two-week acclimation period, another female mouse was co-housed 2 weeks, 1 week, or 24 h to create a stimulation mouse. Environmental conditions were maintained at 22–23°C, 40–60% relative humidity, and a 12-h light/dark cycle (lights on at 07:00). We conducted the experiment under light phase, with the duration ranging from 1 p.m. to 5 p.m. Food and water were available ad libitum.

### 2.2 Behavioral testing

Before the behavioral test, mice were habituated 2 weeks to the experimenter to reduce stress and promote adaptation. Habituation occurred every day in the first week, and every other day in the second week. During habituation sessions, the experimenter wore latex gloves to familiarize the mice with human handling. In the initial phase, mice were gently held by the midtail and allowed to move freely on the gloved hand until they exhibited no signs of anxiety, such as jumping, tail erection, urination, defecation and voluntarily approached the glove. On the test day, mice were allowed at least 1 h to acclimate to the testing environment before experiments began. The stimulus mice were given prior experience with the cup before testing. They were placed in the cup for 10 min daily for three consecutive days to ensure familiarity with the environment. The lighting level in the three-chamber box was maintained at 30 lux.

#### 2.2.1 Classic three-chamber test

The three-chamber apparatus consisted of a rectangular, coverless acrylic box divided into three equalsized compartments (20 cm × 40 cm × 20 cm), with a central passage allowing free movement of the mice. The metal cups (upper ring diameter of 100 mm, lower ring diameter of 80 mm, height of 106 mm) were supplied by Nanyang Wanxiang Biotechnology Co., Ltd. The classic three-chamber social test for both male and female mice was conducted as previously described (Kaidanovich-Beilin et al., 2011; Pan et al., 2022). The test comprised three phases: habituation, sociability test (Stage2), and social preference test (Stage3).

In habituation, the mouse was placed in the central compartment and allowed to explore all three compartments for 10 min. During sociability test, a novel, age- and sex-matched C57BL/6 J mouse was placed in one of the metal cups in either the left or right compartment, with an empty metal cup placed in the opposite compartment. The mouse was allowed to explore for 10 min. In social preference test, a second unfamiliar mouse was placed in the opposite metal cup, and the test mouse was allowed to explore again for 10 min. To minimize odor contamination, the apparatus and cups were wiped with 75% ethanol between trials. All behaviors were recorded using the Any-maze video tracking system (Stoelting Co.).

#### 2.2.2 New three-chamber test

Eight-week-old female C57BL/6J mice were housed in groups of 4–6 per cage for 2 weeks. The habituation stage was same as the three-chamber test. In the sociability stage, a randomly selected female mouse that had been housed with the test mouse for 2 weeks was placed under a metal cup on one side of the chamber as a social stimulus. The opposite side contained an empty metal cup as a nonsocial stimulus. The test mouse was allowed to freely explore for 10 min. In the social preference stage, an unfamiliar female mouse (age-matched C57BL/6J mouse) that had not been housed with the test mouse was placed under the metal cup on the other side. The two metal cups were symmetrically placed, and the test mouse was allowed to explore for an additional 10 min.

For the 1 week or 24 h cagemate test, two unfamiliar female mice (age-matched C57BL/6J mice) were housed in the home cage 1 week or 1 day. On the day of testing, the same procedure as described previously was followed for the social interaction test. The experimental apparatus was wiped with 75% ethanol solution between trials. Social behavioral changes were recorded using the Any-maze video tracking system (Stoelting Co.).

#### 2.2.3 Behavioral data analysis

All data collection and analysis were performed in a double-blind manner. Behavioral data were manually recorded using XNote Stopwatch software, and the interaction time between the mice and the metal cage was quantified. We meticulously assessed a range of behaviors indicative of social engagement, including directly sniffing the mice inside the metal cup and the exposed tails of the stimulus mice, with the mouse’s head located within 2 cm of the outer edge of the metal cup, climbing on the cup, and running around the metal cup. The preference index for the social interaction test was calculated as the ratio of time spent exploring the metal cage containing the mouse versus the time spent exploring the empty metal cage. The preference index for the social recognition test was calculated as the ratio of time spent exploring the metal cage containing the completely unfamiliar mouse versus the time spent exploring the cage containing the mouse from the previous phase.

### 2.3 Statistics

All statistical analyses were performed using Prism 9 (GraphPad) software, and the results are presented as mean ± SEM. Box plots were displayed with the median line, the upper and lower boundaries representing the interquartile range, and the whiskers representing the maximum and minimum values. All data were first tested for normality before statistical testing. Statistical significance was determined using unpaired *t*-test, paired *t*-test, or one-way ANOVA, for non-normally distributed data, the Mann Whitney *U* test or Wilcoxon signed-rank test as appropriate. A *p* < 0.05 was considered statistically significant (*p < 0.05, **p < 0.01, ***p < 0.001).

## 3 Results

### 3.1 Male mice exhibit a preference for novel mice

**Figure 1:**
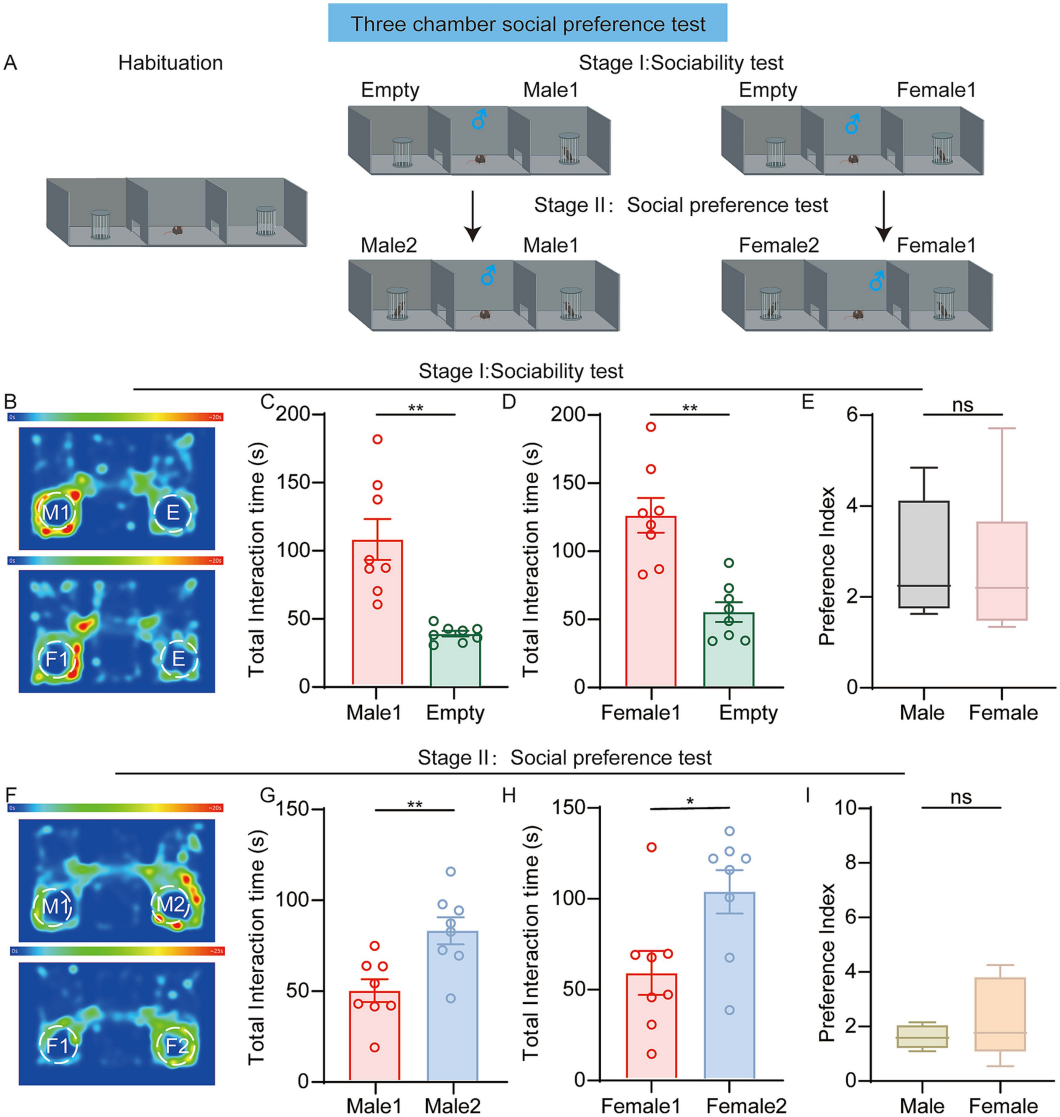
**(A)** Diagram showing the three-chamber test for male mice. **(B–E)** Sociability test. **(B)** Representative heatmaps. The labels “M1,” “F1” indicate male1 and female1, while “E” denotes the empty chamber. Up: male mice socializing with male1 or the empty chamber. Down: male mice socializing with female1 or the empty chamber. **(C)** Time spent (male: *n* = 8 mice, *p* = 0.0029; paired *t*-test) exploring the male1 chamber or the empty chamber. **(D)** Time spent (male: *n* = 8 mice, *p* = 0.0027; paired *t*-test) exploring the female1 chamber or the empty chamber. **(E)** Preference index (*p* = 0.8323; Unpaired *t*-test). Values represent the ratio of time spent investigating the social stimulus relative to the empty chamber. **(F–I)** Social preference test. **(F)** Representative heatmaps. The labels “M2,” “F2” indicate male2 and female2. Up: male mice socializing with male1 or male2. Down: male mice socializing with female1 or female2. **(G)** Time spent (male: *n* = 8 mice, *p* = 0.0045; paired *t*-test) exploring the male1 chamber or the male2 chamber. **(H)** Time spent (male: *n* = 8 mice, *p* = 0.0362; paired *t*-test) exploring the female1 chamber or the female2 chamber. **(I)** Preference index (*p* = 0.9591; Mann–Whitney test). Values represent the ratio of time spent investigating mouse2 relative to mouse1. *p < 0.05; **p < 0.01; **p < 0.001; ns, not significant. Data are presented as mean ± SEM.

We first investigated the performance of 10-week-old male mice in the classic three-chamber social test. During the sociability test stage, each male mouse demonstrated a significant preference for exploring the novel mouse (Male1) over the empty cup (Figures 1A,B top,C). Similarly, when the male social object was replaced with a female one, the male mice consistently spent more time interacting with the unfamiliar female (Female1) compared to the empty cup (Figures 1B bottom,D). In the social preference test stage, when exposed to a novel male mouse (Male2) and a previously encountered male mouse 1 (familiar), the test male mouse spent significantly more time exploring the novel mouse compared to familiar mouse (Figures 1F top,G). In another set of experiment, when presented a novel female mouse (Female2) and the familiar female1, the test male mouse continued to show a preference for the novel female mice (Figures 1F bottom,H). The social preference indices of subject mice toward male and female conspecifics showed no statistical difference. (Figures 1E,I). These findings are consistent with previous studies, confirming that male mice exhibit a preference for conspecifics over an empty cage and novel mice over the familiar ones.

### 3.2 Female mice exhibit no significant preference for novel mice

**Figure 2:**
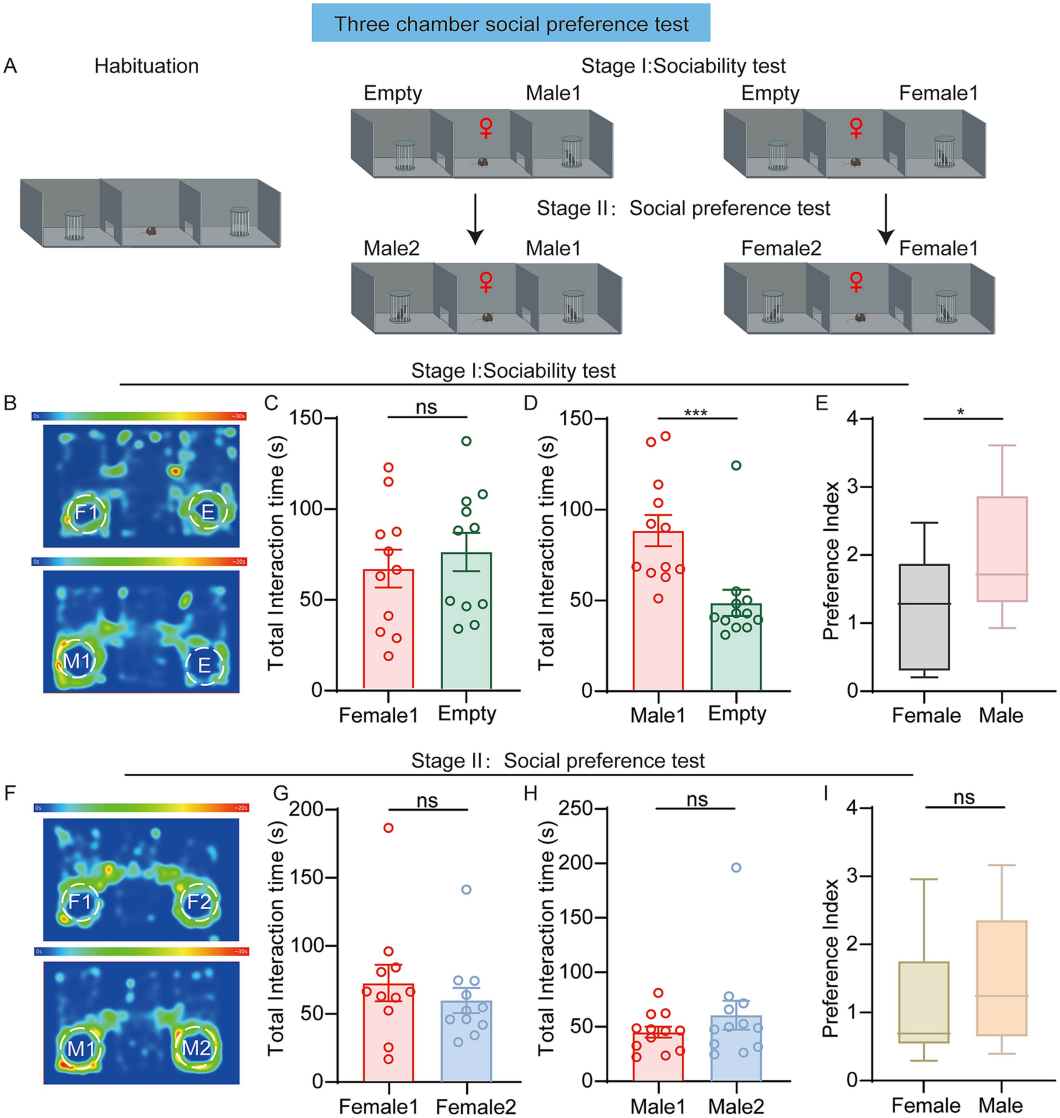
**(A)** Diagram showing the three-chamber test for female mice. **(B–E)** Sociability test. **(B)** Representative heatmaps. The labels “M1” and “F1” refer to Male1 and Female1, and “E” indicates the empty chamber. Top: Female mice exploring the female1 or the empty chamber. Bottom: Female mice exploring the male1 or the empty chamber. **(C)** Time spent (female: *n* = 11 mice, *p* = 0.5603; paired *t*-test) exploring the female1 chamber or the empty chamber. **(D)** Time spent (female: *n* = 12 mice, *p* = 0.0010; Wilcoxon test) exploring the male1 chamber or the empty chamber. **(E)** Preference index (*p* = 0.0184; Unpaired *t*-test). Values represent the ratio of time spent investigating the social stimulus relative to the empty chamber. **(F–I)** Social preference test. **(F)** Representative heatmaps. The labels “M2” and “F2” refer to Male2 and Female2. Top: Female mice exploring the female1 or female2. Bottom: Female mice exploring the male1 or male2. **(G)** Time spent (female: *n* = 11 mice, *p* = 0.7002; Wilcoxon test) exploring the female1 chamber or the female2 chamber. **(H)** Time spent (female: *n* = 12 mice, *p* = 0.4238; Wilcoxon test) exploring the male1 chamber or the male2 chamber. **(I)** Preference index (*p* = 0.3164; Mann–Whitney test). Values represent the ratio of time spent investigating mouse2 relative to mouse1. *p < 0.05; **p < 0.01; **p < 0.001; ns, not significant. Data are presented as mean ± SEM.

We next assessed the performance of 10-week-old female mice in sociability test and social preference test. In the sociability test stage, each female mice were presented with an unfamiliar female mouse (Female1) and an empty cup. Contrasting with male behavior, female mice distributed their exploration time equally between novel female mice and empty cups (Figures 2A,B top, C), demonstrating no significant social preference. However, when the test female mouse was presented with an unfamiliar male mouse (male1) and an empty cup, the female mice spent more time exploring the male1 (Figures 2B bottom,D). The social preference index for male mouse was significantly higher compared to the female mouse (Figure 2E). These results suggested that although female mice preferred to interact with unfamiliar male mice, they exhibit no preference for unfamiliar female mice in the classic three-chamber social test.

In the social preference test stage, the female mice were presented with another novel female mouse (Female2) and the previously encountered female1 (familiar). However, the time spent exploring female2 and female1 did not differ significantly (Figures 2F top,G). Similarly, there was no significant difference in exploration time between male1 (familiar) and male2 (novel) (Figure 2F bottom,H). In this stage, the social preference index for the female mice toward either female mice or male mice showed no significant difference (Figure 2I). These results suggest that female mice did not display a social interest to novel conspecifics in the classic three-chamber social paradigm.

### 3.3 Female mice prefer novel mice to cagemates

**Figure 3:**
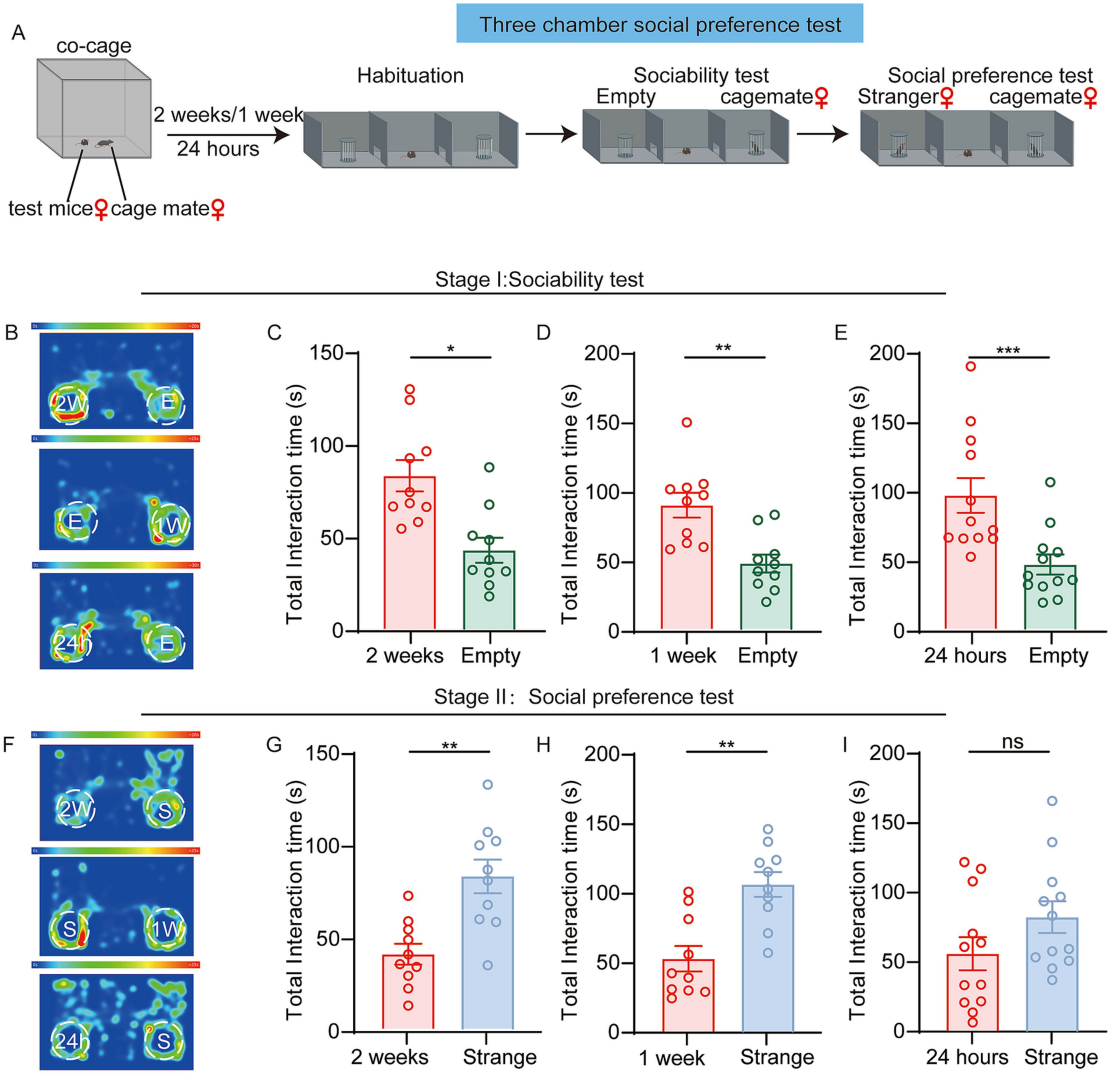
**(A)** Diagram illustrating the new three-chamber test for female mice interacting with cagemate-2 weeks, cagemate-1 week or cagemate-24 h. **(B–E)** Sociability test. **(B)** Representative heatmaps. “2 W”, "1 W”and “24 h” denote the cagemate co-housed for 2 weeks, 1 week or 24 h, “E” denotes the empty chamber. Top: Female mice socializing with the cagemate-2 weeks or the empty chamber. Middle: Female mice socializing with the cagemate-1 week or the empty chamber. Bottom: Female mice socializing with the cagemate-24 h or the empty chamber. **(C)** Time spent (female mice: *n* = 10, *p* = 0.0131; paired *t*-test) exploring the cagemate-2 weeks chamber or the empty chamber. **(D)** Time spent (female mice: *n* = 10, *p* = 0.0011; paired *t*-test) exploring the cagemate-1 week chamber or the empty chamber. **(E)** Time spent (female mice: *n* = 12, *p* = 0.0005; Wilcoxon test) exploring the cagemate-24 h chamber or the empty chamber. **(F–I)** Social preference test. **(F)** Representative heatmaps. “S” indicates the stranger mouse. Top: Female mice socializing with the cagemate-2 weeks chamber or stranger. Middle: Female mice socializing with the cagemate-1 week chamber or stranger. Bottom: Female mice socializing with the cagemate-24 h stranger. **(G)** Time spent (female mice: *n* = 10, *p* = 0.0022; paired *t*-test) exploring the cagemate-2 weeks or the stranger chamber. **(H)** Time spent (female mice: *n* = 10, p = 0.002; Wilcoxon test) exploring the cagemate-1 week or the stranger chamber. **(I)** Time spent (female mice: *n* = 12, *p* = 0.0933; paired *t*-test) exploring the cagemate-24 h chamber or stranger chamber. *p < 0.05; **p < 0.01; ***p < 0.001; ns, not significant. Data are presented as mean ± SEM.

To establish a reliable paradigm capable of detecting social behavior in female mice, we optimized the classic three-chamber social paradigm by replacing one of the social objects with a sex matched cagemate in the sociability stage. The test female mice and the social stimuli female mice had been housed together for either 2 weeks, 1 week or 24 h (Figure 3A). In the sociability test stage, female mice exhibited a significant preference for cagemate (cagemate-2w, cagemate-1w and cagemate-24 h) over an empty cup (Figures 3B–E).

In the social preference test stage, female mice demonstrated significant novelty-seeking behavior, spending more time investigating the novel female (Female2) than the familiar cagemate-2w and cagemate-1w (Figures 3F top and middle,G,H). However, when presented with a cagemate-24 h versus a novel female, this preference pattern showed a non-significant trend toward novelty exploration (Figures 3F bottom,I). These findings demonstrate that substituting a familiar cagemate for a standard social stimulus induces robust novelty-seeking behavior in female mice, with significant preferential exploration directed toward novel females.

### 3.4 Total social interaction time showed no paradigm-dependent variation

**Figure 4:**
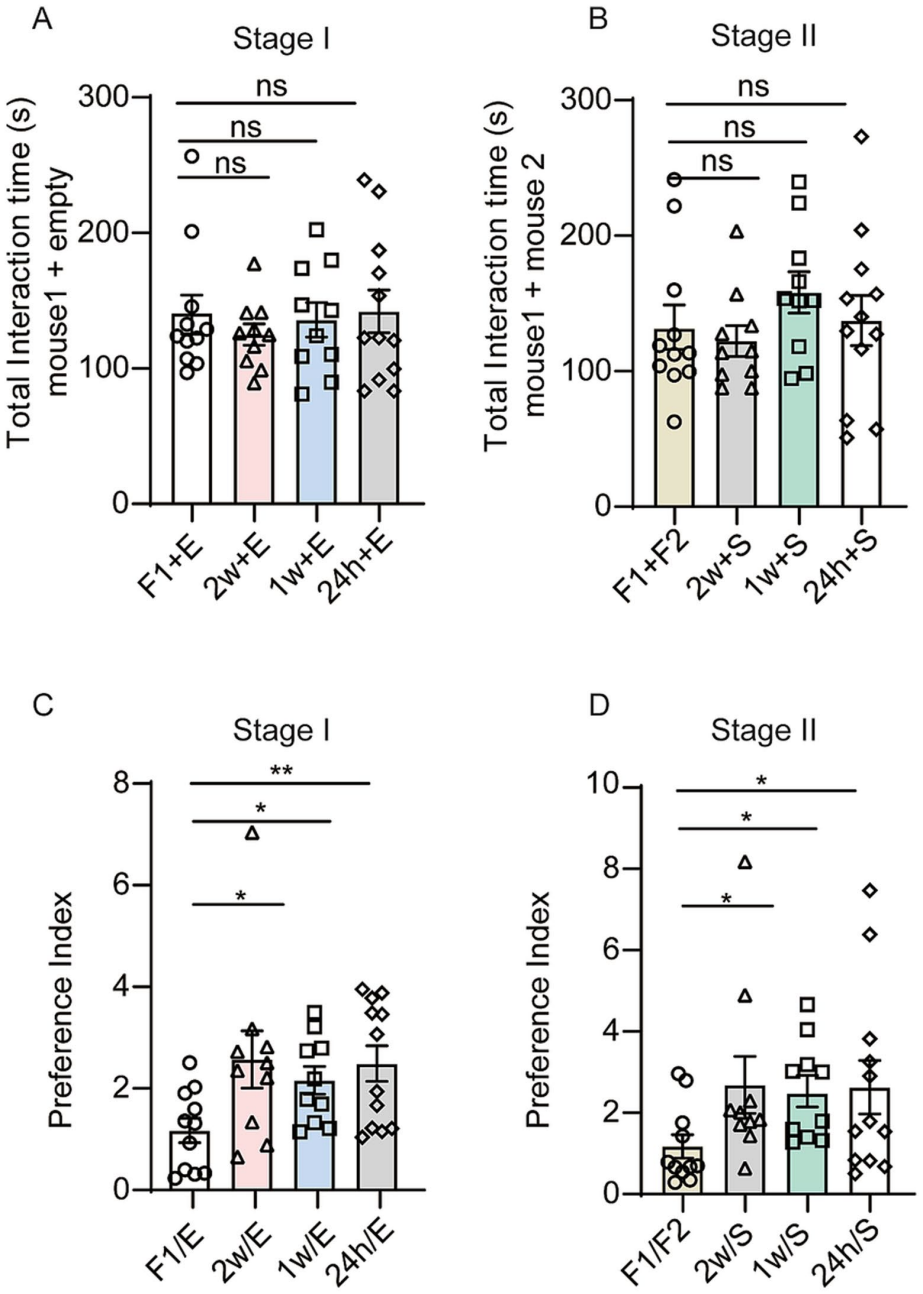
**(A,B)** Total interaction time. The labels “F1,” “F2” indicate female1 and female2, “2 W”, "1 W”and “24 h” denote the cagemate co-housed for 2 weeks, 1 week or 24 h, “E” denotes the empty chamber, “S” indicates the stranger mouse, “+” indicates the plus. **(A)** Sociability test. Total interaction time of the female test mouse spent with female1 and empty cup compared with a cagemate 2 weeks and empty cup (*p* = 0.5868), a cagemate 1 week and empty cup (*p* = 0.9445), a cagemate 24 h and empty cup (*p* = 0.7847). **(B)** Social preference test. Total interaction time of the test female mouse spent with female2 and female1 compared with a strange mouse and a cagemate 2 weeks mouse (*p* = 0.7541), a strange mouse and a cagemate 1 week mouse (*p* = 0.1785), a strange mouse and a cagemate 24 h mouse (*p* = 0.5544). **(C,D)** Preference index. “/” indicates the divide. **(C)** Sociability test. Preference index of classic three-chamber female mice test group compared with the cagemate 2 weeks (*p* = 0.0253), cagemate 1 week (*p* = 0.0417) and cagemate 24 h (*p* = 0.0090). **(D)** Social preference test. Preference index of classic three-chamber female mice test group compared with the cagemate 2 weeks (*p* = 0.0155), cagemate 1 week (*p* = 0.0147) and cagemate 24 h (*p* = 0.0416). One-way ANOVA for **(A–D)**. *p < 0.05; ns, not significant. Data are presented as mean ± SEM.

It is important to note that in our new three-chamber social test paradigm, the social preference of female mice in both phases could be altered. Total social interaction time showed no paradigm dependent variation, with equivalent measurements between the novel and traditional three-chamber designs, indicating preserved core behavioral metrics across test configurations (Figures 4A,B). However, the social preference indices in both phases were significantly higher in our new social test paradigm than those observed in the classic three-chamber test (Figures 4C,D), suggesting that we successfully developed a novel behavioral paradigm that accurately quantifies female specific social patterns while maintaining baseline social interaction duration.

## 4 Discussion

The classic three-chamber social paradigm is effective for assessing social preference in male mice but shows limited reliability for evaluating female social behaviors. To address this, we established a female adapted new three-chamber social paradigm. When exposed to familiar cagemates, female mice demonstrate enhanced social exploration and novel-social preference, revealing more complex integration of sex and familiarity cues compared to males. This paradigm provides an essential framework for studying social behavior patterns and social deficits in female autistic mice.

When encountering a distressed companion, female mice display social approach behavior resembling consolation, while male mice exhibit anxiety like self-grooming behavior serving a self-consolation function (Fang et al., 2024). Long term social isolation intensifies aggressive behavior in male mice but show no measurable effect on female aggressive behaviors (Wang et al., 2022). Consistent with prior studies, substantial evidence shows female mice behave differently from male mice in the social test (Moy et al., 2004; Karlsson et al., 2016; Bertoni et al., 2021). During the sociability stage of the classic paradigm, male mice show significant preference for both unfamiliar female and male mice. This may stem from male mice’s territoriality and social hierarchy (Zheng et al., 2025). Male mice explore to assess the strength and threat of unfamiliar males. Their preference for unfamiliar females could be driven by sexual and reproductive instincts, as female-derived pheromones constitute potent chemosensory cues in murine social communication. In this stage, female mice demonstrate null preference for unfamiliar females and clear preferential interaction with novel male conspecifics, potentially influenced by their virgin status enhancing chemosignal sensitivity (Roberts et al., 2010), while possibly acquiring survival benefits through male-mediated group protection (Wei et al., 2025).

During the social preference test, male mice spend more time exploring novel conspecifics compared to familiar ones. This novelty-driven exploration may be mediated by pheromonal divergence in unfamiliar individuals, which could engage dopaminergic reward pathways to induce pleasure. However, female mice showed no significant preference for novel conspecifics in this stage. Rather than indicating an inability to differentiate social familiarity, we proposed that female social behavior involves multifactorial valuation requiring integration of familiarity-novelty cues. This complexity fundamentally limits the classic three-chamber paradigm’s utility in evaluating female social behaviors.

The mesolimbic dopaminergic system is central to the social reward (Dai et al., 2022; Solié et al., 2022; Bayless et al., 2023; Flannery et al., 2023; Blum et al., 2024). During social exploration, dopamine system exhibits adaptive plasticity based on social context, species cues, and familiarity states (Dai et al., 2022; Molas et al., 2024). In adult female mice, the social reward effect demonstrates context dependent heterogeneity rather than universal responsiveness. Female mice may experience a stronger reward when interacting with familiar companions in the sociability test. The presence of a familiar cagemate may establish a security priming effect, enabling novel-social exploration in female mice.

It is important to note that in the current study we only used one strain of mice - C57BL/6 J and did not consider the estrous cycle of the female mice. It has been suggested that nonreceptive stages (metestrus and diestrus) females are able to distinguish between empty chamber and the stranger mouse, while sexually-receptive stages (proestrus and estrus) females show no difference between them (Chari et al., 2020). Besides, different strains exhibit distinct genetic, behavioral, and physiological profiles. These differences may affect the social behavior. Mice from three inbred strains, C57BL/6J, DBA/2J, FVB/NJ, and B6129PF2/J hybrid mice showed significant sociability and preference for social novelty. In contrast, A/J inbred mice do not exhibit these behaviors to the same extent (Moy et al., 2004). Whether our paradigm can effectively detect sociability in female mice across all estrous phases and various strains warrants further exploration.

Overall, our study demonstrates that the classic three-chamber social paradigm lacks reliability in assessing female social preferences. When exposed to familiar cagemates, female mice exhibit novel social stimulus preference a behavioral dichotomy not captured by traditional paradigms. These adaptations establish a validated framework for investigating female social behavior, addressing the persistent sex bias in preclinical behavioral neuroscience.

## Data Availability

The original contributions presented in the study are included in the article/supplementary material, further inquiries can be directed to the corresponding author.
